# CC-90009, a Cereblon E3 Ligase Modulator, Exhibits Antiviral Efficacy Against JEV In Vitro and In Vivo via Targeted Degradation of GSPT1 and Viral NS5 Protein

**DOI:** 10.3390/pharmaceutics17121524

**Published:** 2025-11-27

**Authors:** Zhiwei He, Yibo Chen, Binghui Xia, Zimeng Cheng, Ping Zhao, Zhongtian Qi, Yongzhe Zhu

**Affiliations:** 1 Department of Microbiology, Faculty of Naval Medicine, Naval Medical University, Shanghai 200433, China; hezw@smmu.edu.cn (Z.H.); miraclechen03@163.com (Y.C.); xbhnjucpu@smmu.edu.cn (B.X.); 13582818881@163.com (Z.C.); pnzhao@163.com (P.Z.); 2 Shanghai Key Laboratory of Medical Bioprotection, Second Military Medical University, Shanghai 200433, China; 3 Key Laboratory of Biological Defense, Ministry of Education, Second Military Medical University, Shanghai 200433, China

**Keywords:** Japanese encephalitis virus (JEV), cereblon E3 ligase modulators (CELMoD), broad-spectrum antiviral activity, G1-to-S phase transition 1 (GSPT1), non-structural proteins

## Abstract

**Background:** Japanese encephalitis virus (JEV), a mosquito-borne flavivirus, remains a leading cause of viral encephalitis. Current management is largely supportive, with no specific antivirals. This study evaluated the antiviral efficacy and mechanism of action of CC-90009 against JEV in vitro and in vivo. **Methods:** Five targeted protein degraders (TPDs) were screened for anti-JEV activity in the human neuroblastoma cell line SH-SY5Y. Time-of-addition, binding, and endocytosis assays were used to delineate the phase of action of CC-90009, a cereblon (CRBN) E3 ligase modulator (CELMoD) and molecular glue degrader. Small interfering RNA knockdown and co-immunoprecipitation (Co-IP) confirmed targets essential for its antiviral effects. The broad-spectrum activity of CC-90009 against other mosquito-borne viruses was also evaluated. In vivo efficacy was tested in a murine JEV model. **Results:** Of the five TPDs tested, only CC-90009 significantly inhibited JEV infection in SH-SY5Y cells, acting during both viral entry and post-entry phases without reducing adsorbed or internalised virions. CC-90009 reduced JEV RNA and non-structural protein accumulation. Knockdown of G1-to-S phase transition 1 (GSPT1), a key target of CC-90009, suppressed JEV infection and translation; Co-IP confirmed GSPT1 interaction with JEV non-structural protein 5 (NS5). CC-90009 disrupted JEV translation and replication by inducing proteasomal degradation of the GSPT1/NS5 complex, further demonstrating its broad-spectrum antiviral activity through the effective inhibition of West Nile virus and chikungunya virus. In vivo, it protected mice from JEV-induced mortality, reducing viral load, antigen levels, and brain pathology. **Conclusions:** CC-90009 exerts potent anti-JEV activity both in vitro and in vivo by inducing proteasomal degradation of the GSPT1/NS5 complex, thereby disrupting viral translation and replication. This targeted protein degradation strategy represents a novel host-directed antiviral approach with promising therapeutic potential against mosquito-borne viral encephalitis.

## 1. Introduction

Japanese encephalitis (JE) is an acute infectious disorder of the central nervous system resulting from infection with Japanese encephalitis virus (JEV), which belongs to the Flaviviridae family [1]. JEV is transmitted between animal and human hosts via mosquitoes of the *Culex* genus [2]. As a significant human-pathogenic virus, it is responsible for approximately 100,000 cases of acute encephalitis annually. Among confirmed cases, the case fatality rate ranges from 25% to 40%, while persistent neurological complications are observed in as many as half of all survivors [3]. Despite the availability of vaccines, JEV remains a significant public health concern and retains the potential for global dissemination [4]. At present, no specific antiviral medications are available for JE, and clinical management is centred on symptomatic treatment. As a result, there is an urgent requirement to discover new antiviral candidates with optimal safety and therapeutic efficacy against JEV infection.

JEV is a single-stranded positive-sense RNA virus with a genome of approximately 11 kilobases [5]. It encodes 10 viral proteins, comprising three structural proteins (core, premembrane, and envelope) and seven non-structural proteins (NS1, NS2A–B, NS3, NS4A–B, and NS5) [6]. The process of JEV infection in nerve cells involves multiple stages. Initially, the virus attaches to and enters host cells via unidentified host receptors, followed by caveolin-mediated endocytosis [7]. Once in the cytoplasm, the viral RNA commences translation and replication [8]. A key function of the non-structural proteins is to form the viral replication complex, facilitating critical processes such as genome replication, virion assembly, and evasion of host immune responses through interactions with host cell proteins [9]. JEV NS5 is a multifunctional, evolutionarily conserved protein, and its RNA-dependent RNA polymerase (RdRp) activity is crucial for replication of the viral genome [10]. Consequently, NS5 has emerged as a pivotal antiviral target for therapeutic development.

Emerging targeted protein degradation (TPD) technologies, notably proteolysis-targeting chimeras (PROTACs) and molecular glue degraders, act by binding to protein–protein interaction interfaces, inducing spatial proximity to drive target protein degradation via the ubiquitin–proteasome system [11]. Clinical-stage compounds, including cereblon (CRBN) E3 ligase modulators (CELMoDs)—a class of molecular glue degraders—such as CC-92480 [12], NVP-DKY709 [13], and CC-90009 [14], as well as PROTACs such as ARV-110 [15] and ARV-471 [16], have shown promising antitumour efficacy. Their mechanisms of action against mosquito-borne viral infections, including JEV, however, remain poorly characterized. Unlike conventional small-molecule inhibitors, which fail to regulate target protein abundance, struggle with “undruggable” pocketless targets, require high doses, and frequently induce resistance, TPD leverages the host’s protein degradation machinery for selective clearance of pathogenic proteins, offering advantages in reducing toxicity, overcoming resistance, and targeting challenging substrates [17].

CC-90009 is a CELMoD that functions as a molecular glue degrader. It binds to CRBN, the substrate receptor of the Cullin4-RING E3 ubiquitin ligase complex (CRL4), thereby targeting G1-to-S phase transition 1 (GSPT1) for ubiquitination and subsequent degradation via the proteasome [18]. CC-90009 is in Phase I trials to assess its efficacy and safety in patients with acute myeloid leukaemia. Recent studies have indicated that CC-90009 exhibits antiviral activity against both Lassa virus (LASV) [19] and Ebola virus (EBOV) [20] without significant cytotoxicity. Prompted by these findings, we sought to evaluate the antiviral potential of CC-90009 against JEV using the human neuroblastoma cell line SH-SY5Y and a suckling mouse model of infection. Our findings demonstrate that CC-90009 markedly suppresses JEV infection in vitro and confers protection in vivo against JEV-induced mortality, supporting its potential for repurposing as a broad-spectrum antiviral agent against mosquito-borne viral infections.

## 2. Materials and Methods

### 2.1. Cells and Viruses

SH-SY5Y (ATCC CRL-2266), baby hamster kidney cell line BHK-21 (ATCC CCL-10), African green monkey kidney Vero cells (ATCC CCL-81), and human embryonic kidney cell line HEK-293T (ATCC CRL-11268) were maintained in Dulbecco’s modified eagle medium (Thermo Fisher Scientific, Waltham, MA, USA) supplemented with 10% foetal bovine serum (GIBCO, Waltham, MA, USA) at 37 °C in a 5% CO_2_-humidified incubator. The JEV strain SA14 (GenBank accession no. U14163.1) was propagated and titrated on BHK-21 cells, with viral titres quantified using a plaque assay. Chikungunya virus (CHIKV) infectious clone of Ross isolate (GenBank accession no. AF490259) and West Nile virus (WNV) infectious clone of NY2000 strain (GenBank accession no. AF404756) were synthesised in this laboratory. CHIKV and WNV were propagated in Vero cells.

### 2.2. Chemicals and Antibodies

CC-90009 (HY-130800), CC-92480 (HY-129395), NVP-DKY709 (HY-144998), ARV-110 (HY-138641), ARV-471 (HY-138642), chloroquine (CQ, HY-17589A), heparin (HY-17567), nystatin (HY-17409), and MG132 (HY-13259) were purchased from MedChemExpress (Monmouth Junction, NJ, USA). The chemical structure and pharmacological profile of CC-90009 are provided in the Supplementary Materials (Figure S1). The Cell Counting Kit-8 was obtained from Invitrogen (Carlsbad, CA, USA). Antibodies against JEV NS1 (GTX633820), NS2B (GTX125972), NS3 (GTX125868), NS4A (GTX132028), NS4B (GTX125865), NS5 (GTX131359), as well as CHIKV nsP4 (GTX638958) and WNV NS5 (GTX131961), were purchased from GeneTex (Irvine, CA, USA). Anti-GSPT1 protein (ab49878) and CRBN (ab315344) antibodies were obtained from Abcam (Cambridge, UK). Anti-Ubiquitin antibody (#43124) was obtained from Cell Signaling Technology (Danvers, MA, USA). Alexa Fluor^TM^ 488 secondary antibody was purchased from Invitrogen (Carlsbad, CA, USA).

### 2.3. Small Interfering RNAs (siRNAs), Plasmids and Transfection

Transfection assays were conducted as previously described [21]. Cells (50–60% confluence) were transfected with 80 nM siRNA or 800 ng plasmid and incubated for 48 h at 37 °C. The siRNAs targeting GSPT1 and CRBN were purchased from GenePharma (Shanghai, China). Plasmids encoding human GSPT1, JEV NS5, WNV NS5 and CHIKV nsP4 were obtained from GeneChem (Shanghai, China).

### 2.4. Immunofluorescence (IF) Assay

Following methanol fixation, infected cells were incubated in a blocking solution containing 3% bovine serum albumin for 2 h. Immunofluorescence staining was performed using the following primary antibodies, incubated overnight at 4 °C: a rabbit anti-JEV NS3 antibody (GTX125868), a rabbit anti-CHIKV nsP4 antibody (GTX638958), and a rabbit anti-WNV NS5 antibody (GTX131961). Subsequently, the cells were incubated with an Alexa Fluor™ 488-conjugated goat anti-rabbit immunoglobulin G (IgG) secondary antibody for 1 h at 25 °C. Finally, cells were stained with phosphate-buffered saline containing 0.1 µg/mL 4′,6-diamidino-2-phenylindole for 10 min before imaging. All immunofluorescence results were acquired using the BioTek Lionheart FX Imaging Reader (Agilent, Santa Clara, CA, USA), and the number of positive cells across the entire well was quantified.

### 2.5. Quantitative Real-Time Polymerase Chain Reaction (qRT-PCR)

To quantify viral RNA levels, total RNA was extracted from treated SH-SY5Y cells or mouse brains using TRIzol reagent (TaKaRa, Shiga, Japan). First-strand cDNA synthesis was performed using PrimeScript RT Master Mix (TaKaRa) according to the manufacturer’s instructions. PCR amplification of JEV RNA and glyceraldehyde 3-phosphate dehydrogenase (GAPDH; reference gene) was performed with SYBR Premix Ex Taq (TaKaRa) using respective primer pairs in an ABI 7300 system (Applied Biosystems, Foster City, CA, USA). Copy numbers of JEV RNA were normalised to GAPDH levels using comparative cycle threshold values (2^−ΔΔCt^) determined in parallel. The following primer sets were used for amplification. Specifically, the primers were designed against the 3′ untranslated region of the JEV SA14 strain (GenBank accession number: U14163.1).

JEV: forward 5′-CCCTCAGAACCGTCTCGGAA-3′, reverse 5′-CTATTCCCAGGTGTCAATATGCTGT-3′;

GAPDH: forward 5′-TGGGCTACACTGAGCACCAG-3′, reverse 5′-AAGTGGTCGTTGAGGGCAAT-3′.

### 2.6. Western Blotting

The assay was conducted as previously described [22]. Following collection in RIPA buffer (Thermo Fisher Scientific), cells were lysed by sonication and denatured by boiling. Protein samples were normalized by concentration, resolved by 10% SDS-PAGE, and electrotransferred onto PVDF membranes (Bio-Rad, Hercules, CA, USA). Subsequently, the membranes were blocked with 5% skimmed milk for 2 h at room temperature prior to incubation with the specified primary and HRP-conjugated secondary antibodies. Protein bands were visualised with the ECL Plus enhanced chemiluminescence detection reagents (PerkinElmer Life Sciences, Waltham, MA, USA), depending on signal intensity.

### 2.7. Co-Immunoprecipitation (Co-IP)

The assay was conducted as previously described [23]. At 48 h post-transfection with the indicated plasmids, cells were harvested from 6-well plates. Cells were lysed on ice for 4 h using a lysis buffer composed of 50 mM Tris (pH 7.4), 150 mM NaCl, 1 mM EDTA, 1% Triton X-100, 10% glycerol, 10 µg/mL leupeptin, 10 µg/mL aprotinin, and 2 mM PMSF. Following centrifugation of cell lysates at 13,000 rpm for 10 min, the resulting supernatants were incubated for 1 h with 1 µg of either anti-GSPT1 antibody or a rabbit IgG isotype control (GeneTex, GTX213110-01). Subsequently, 20 µL of protein G agarose beads (Roche, Basel, Switzerland) were added, and the mixture was incubated overnight at 4 °C with gentle rotation. After centrifugation, pellets were washed with lysis buffer, and target proteins were detected by Western blotting using specific antibodies.

### 2.8. Time-Window Compound Assay

To elucidate the stage of the viral life cycle targeted by CC-90009, JEV-infected SH-SY5Y cells (MOI = 0.5) were treated with 10 µM compound at various time points relative to infection, ranging from 4 h pre-infection to 24 h post-infection at 4 h intervals. All infections and treatments were carried out at 37 °C. Viral infection levels were quantified by IF.

### 2.9. Mouse Experiments

One-day-old Institute of Cancer research suckling mice were subcutaneously inoculated with JEV at a dose of 4 × 10^3^ plaque-forming units per mouse. Mice were subsequently administered CC-90009 (20 mg/kg) subcutaneously once daily for five consecutive days. The compound was formulated in a vehicle containing dimethyl sulfoxide (DMSO) and 20% sulfobutylether-β-cyclodextrin (HY-17031, MedChemExpress) in saline (10:90, *v*/*v*). Control-infected mice received the same volume of DMSO and 20% sulfobutylether-β-cyclodextrin. The survival rates were assessed daily until 14 days post-infection. Organs and tissues were collected from euthanised mice at the indicated time points and fixed with 4% paraformaldehyde (Servicebio, Wuhan, China) at 4 °C for 24 h. After fixation, tissues were subjected to histopathological and immunohistochemical analyses.

### 2.10. Statistical Analysis

All data represent the arithmetic mean ± standard deviation (SD) of a minimum of three independent replicates. Two-group comparisons were assessed by Student’s *t*-test, while differences across multiple groups were evaluated using one-way ANOVA with Bonferroni’s correction for post hoc testing, implemented in GraphPad Prism 9.0 (GraphPad Software, San Diego, CA, USA). A *p*-value < 0.05 was considered statistically significant.

## 3. Results

### 3.1. CC-90009 Potently Inhibits JEV Infection in SH-SY5Y Cells

**Figure 1:**
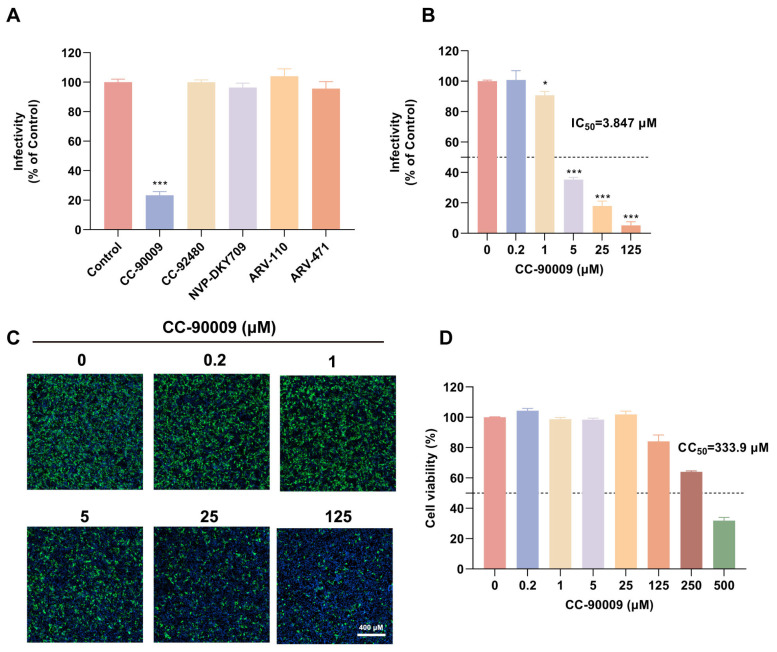
CC-90009 potently inhibits Japanese encephalitis virus (JEV) infection in SH-SY5Y cells. (**A**) SH-SY5Y cells were treated with CC-90009 (10 μM), CC-92480 (5 μM), NVP-DKY709 (10 μM), ARV-110 (5 μM) or ARV-471 (1 μM) and infected with JEV (MOI = 0.5) for 24 h. JEV infectivity was assessed using immunofluorescence (IF). Values were normalised to those of a dimethyl sulfoxide (DMSO) control; (**B**,**C**) SH-SY5Y cells were treated with increasing concentrations of CC-90009 and infected with JEV (MOI = 0.5) for 24 h. Viral inhibition was quantified by IF assay; (**D**) SH-SY5Y cells were incubated with the indicated concentrations of CC-90009 for 24 h at 37 °C, and cell viability was assessed using a cell counting kit-8 assay. Data are shown as means with standard deviation (SD) of three independent experiments. Scale bar, 400 µM. * *p* < 0.05 and *** *p* < 0.001, compared with control.

To investigate the antiviral activity of TPD-based compounds against JEV infection in SH-SY5Y cells, we selected five clinically advanced compounds: CC-90009; CC-92480, a phase III anti-myeloma CELMoD; NVP-DKY709, a phase I anti-tumour CELMoD targeting Ikaros family zinc finger protein 2; ARV-110, a phase II androgen receptor targeting PROTAC; and ARV-471, a phase II oestrogen receptor PROTAC for breast cancer. SH-SY5Y cells were co-treated with JEV and each compound, followed by assessment of their effects on viral infectivity. Screening experiments revealed that CC-90009 significantly inhibited JEV infection in SH-SY5Y cells, whereas the other compounds showed negligible anti-JEV activity (Figure 1A). Further investigations across a broad concentration range confirmed CC-90009′s anti-JEV activity and cytotoxicity. As shown in Figure 1B–D, it exerted a dose-dependent inhibitory effect on JEV infection with a 50% maximal inhibitory concentration (IC_50_) of 3.847 μM and a 50% cytotoxic concentration (CC_50_) of 333.9 μM, highlighting its potential as a promising antiviral agent.

### 3.2. CC-90009 Does Not Affect JEV Binding or Endocytosis

**Figure 2:**
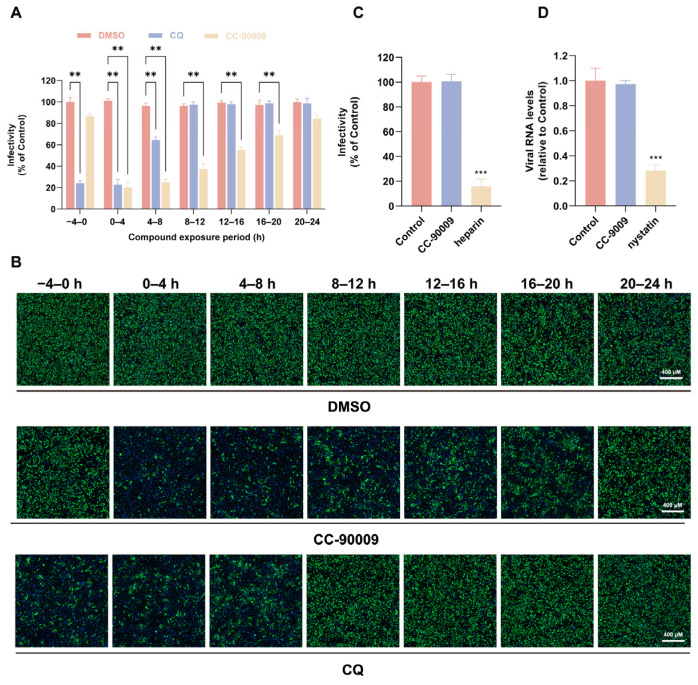
CC-90009 has no effect on JEV binding or endocytosis. (**A**,**B**) SH-SY5Y cells were infected with JEV at a MOI = 0.5 and incubated at 37 °C for 2 h. CC-90009 was administered during the following time windows relative to infection: −4 to 0, 0–4, 4–8, 8–12, 12–16, 16–20, and 20–24 h. Viral infectivity was quantified by IF; (**C**) SH-SY5Y cells were co-incubated with JEV alongside either CC-90009 or heparin for 1.5 h on ice. Following incubation, the cells were washed three times with ice-cold phosphate-buffered saline to remove unbound virions. The cells were subsequently cultured in fresh medium at 37 °C for 24 h to allow infection progression, after which bound virions were assessed by IF; (**D**) Following an initial 1.5 h incubation of SH-SY5Y cells with JEV on ice, the supernatant was replaced with fresh medium containing either CC-90009 or nystatin. The cells were then shifted to 37 °C for 1 h to allow viral internalisation. Following treatment with proteinase K to remove non-internalised virions, intracellular JEV RNA levels were quantified by quantitative real-time polymerase chain reaction (qRT-PCR). Scale bar, 400 µM. ** *p* < 0.01 and *** *p* < 0.001, compared with control.

To identify the stage of the JEV lifecycle inhibited by CC-90009, a time-of-addition assay was performed in which the compound was administered to infected SH-SY5Y cells at various times relative to infection. As shown in Figure 2A,B, compared with CQ, a well-known viral entry inhibitor, CC-90009 exhibited significant suppressive activity across all therapeutic windows except pretreatment, suggesting that CC-90009 might affect both viral entry and post-entry stages. JEV entry mainly comprises binding and endocytosis. We initially examined the role of CC-90009 in the viral binding process. SH-SY5Y cells were co-incubated with JEV and CC-90009 at the indicated concentrations for 1.5 h on ice. The results indicated that CC-90009 did not affect JEV infectivity during the binding stage in SH-SY5Y cells (Figure 2C). In contrast, treatment with the positive control inhibitor heparin resulted in a significant reduction in viral infection. We next investigated whether CC-90009 influences viral endocytosis. It is well established that JEV employs a caveolin-dependent pathway for entry into neuronal cells [7]. Given this established mechanism, we employed RT-qPCR to assess whether CC-90009 impairs JEV entry via this route. Following a 1.5 h incubation of SH-SY5Y cells with the virus on ice, the supernatant was replaced with fresh medium containing CC-90009 prior to a further 1 h incubation at 37 °C. After treatment with proteinase K to remove uninternalised virus, intracellular JEV was quantified. Unlike the positive control nystatin, CC-90009 did not reduce JEV RNA levels, indicating that CC-90009 does not affect viral endocytosis (Figure 2D).

### 3.3. CC-90009 Impairs JEV Translation and Replication

**Figure 3:**
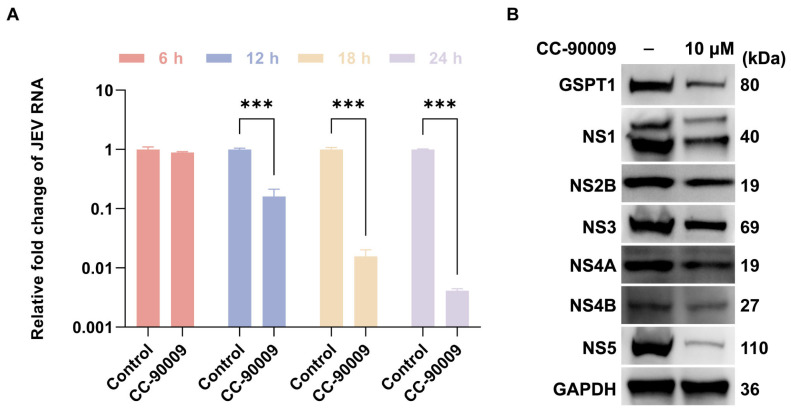
CC-90009 reduces JEV RNA and non-structural protein accumulation. (**A**) SH-SY5Y cells were infected with JEV and treated with CC-90009. JEV RNA levels were measured at the indicated time points; (**B**) SH-SY5Y cells infected with JEV were treated with CC-90009. Levels of G1-to-S phase transition 1 (GSPT1) and JEV non-structural proteins were analysed by Western blot. Values were normalised to those of a DMSO control. *** *p* < 0.001, compared with control.

The above-mentioned results demonstrated that CC-90009 does not affect JEV entry. We therefore investigated whether CC-90009 impacts post-entry stages of JEV infection. Following endocytosis, JEV first translates viral non-structural proteins, which then assemble into replication complexes on the endoplasmic reticulum to facilitate viral RNA replication. To determine if CC-90009 affects JEV translation and replication, SH-SY5Y cells were treated with CC-90009 immediately following JEV infection to evaluate its effects on viral RNA accumulation and non-structural protein expression during a 24 h time course. At 6 h post-infection, CC-90009 mildly decreased JEV RNA accumulation. However, from 12 to 24 h post-infection, CC-90009 significantly reduced JEV RNA accumulation (Figure 3A). CC-90009 also significantly diminished the accumulation of GSPT1 and JEV non-structural proteins compared to the DMSO-treated control at 24 h post-infection. Notably, the inhibitory effect of CC-90009 on NS5 protein levels was the most prominent (Figure 3B).

### 3.4. GSPT1 Facilitates JEV Non-Structural Protein Translation

**Figure 4:**
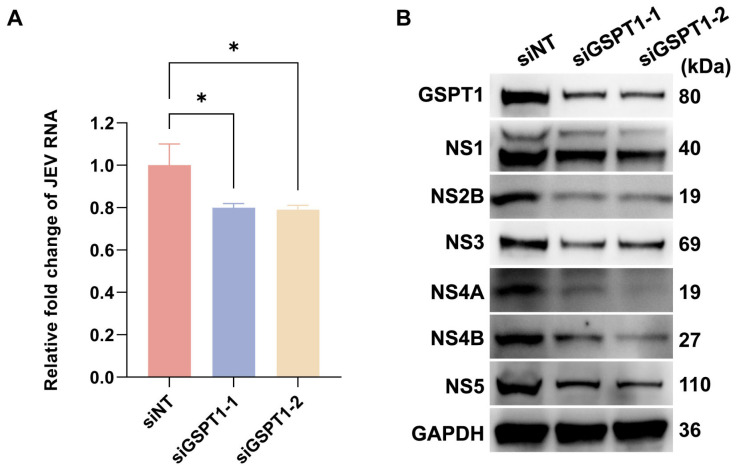
GSPT1 supports the translation of JEV non-structural proteins. (**A**) Quantification by RT-qPCR of JEV RNA levels in SH-SY5Y cells at 12 h post-infection (MOI = 1) following transfection with siGSPT1; (**B**) Protein levels of GSPT1 and JEV non-structural proteins were analysed by Western blot in siGSPT1-transfected cells following JEV infection (MOI = 1). Values were normalised to those of a non-targeting siRNA (siNT) control. * *p* < 0.05, compared with control.

CC-90009 functions as an E3 ubiquitin ligase modulator by selectively recruiting GSPT1 to the CRL4–CRBN complex, leading to its targeted ubiquitination and subsequent degradation. To elucidate the role of GSPT1 in JEV replication and translation, siRNA-mediated knockdown (KD) was employed to assess its impact on both viral RNA accumulation and non-structural proteins expression. Compared with the control group, GSPT1-KD did not significantly suppress JEV RNA levels (Figure 4A), but led to a reduction in the abundance of JEV non-structural proteins (Figure 4B). This finding aligns with the established role of GSPT1 as an essential constituent of the eukaryotic translation termination complex (eukaryotic release factor 1/eukaryotic release factor 3) [24]. Given the subtle impact on JEV RNA accumulation but a decrease in JEV protein levels, we reason that GSPT1 may support JEV infection by facilitating viral protein translation.

### 3.5. CC-90009 Mediates the Degradation of GSPT1 and JEV NS5 via the Proteasome Pathway

**Figure 5:**
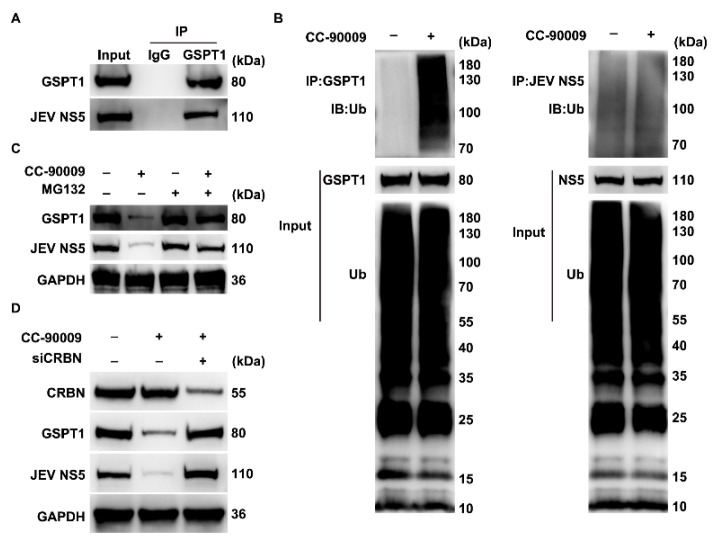
CC-90009 induces proteasome-dependent degradation of GSPT1 and JEV NS5. (**A**) Co-immunoprecipitation (Co-IP) of GSPT1 and JEV NS5 in HEK-293T cells. Lysates were immunoprecipitated with anti-GSPT1 antibody or non-specific immunoglobulin G (IgG) control, followed by Western blot analysis; (**B**) Ubiquitination levels of GSPT1 and JEV NS5 were analysed by Western blot; (**C**) Cells overexpressing GSPT1 and NS5 were treated with CC-90009 or MG132, and protein levels were analysed by Western blot; (**D**) Protein levels were analysed by Western blot following cereblon (CRBN) depletion and CC-90009 treatment.

In contrast to siRNA-mediated GSPT1 depletion, treatment with CC-90009 suppressed the accumulation of JEV RNA following infection, indicating a fundamental defect in JEV RNA synthesis. We initially investigated a potential physical interaction between GSPT1 and the JEV NS5 protein using co-IP. (Figure 5A). Furthermore, treatment with CC-90009 significantly enhanced the ubiquitination of GSPT1, whereas the ubiquitination level of JEV NS5 was unaffected (Figure 5B). Collectively, we hypothesize that CC-90009-mediated tethering of the E3 ligase to GSPT1 may lead to the concomitant degradation of the bound JEV NS5 protein.

To validate this hypothesis, cells overexpressing GSPT1 and NS5 were treated with either CC-90009 or the proteasome inhibitor MG132, and subsequent alterations in protein levels were examined. Treatment with CC-90009 alone reduced the levels of both GSPT1 and NS5, whereas MG132 alone had no significant effect. However, co-treatment with MG132 abrogated the degradation induced by CC-90009, restoring protein levels to those observed with MG132 treatment alone (Figure 5C). Furthermore, CRBN-KD attenuated the CC-90009-induced degradation of both GSPT1 and JEV NS5 (Figure 5D). These results suggest that the degradation of both GSPT1 and NS5 following CC-90009 treatment is mediated by the proteasome pathway. Consequently, diminished levels of JEV NS5 are likely to result in a global suppression of viral RNA synthesis. Collectively, our findings demonstrate that CC-90009 exhibits potent anti-JEV activity in SH-SY5Y cells.

### 3.6. CC-90009 Exerts Broad-Spectrum Antiviral Activity Against Mosquito-Borne Viruses

**Figure 6:**
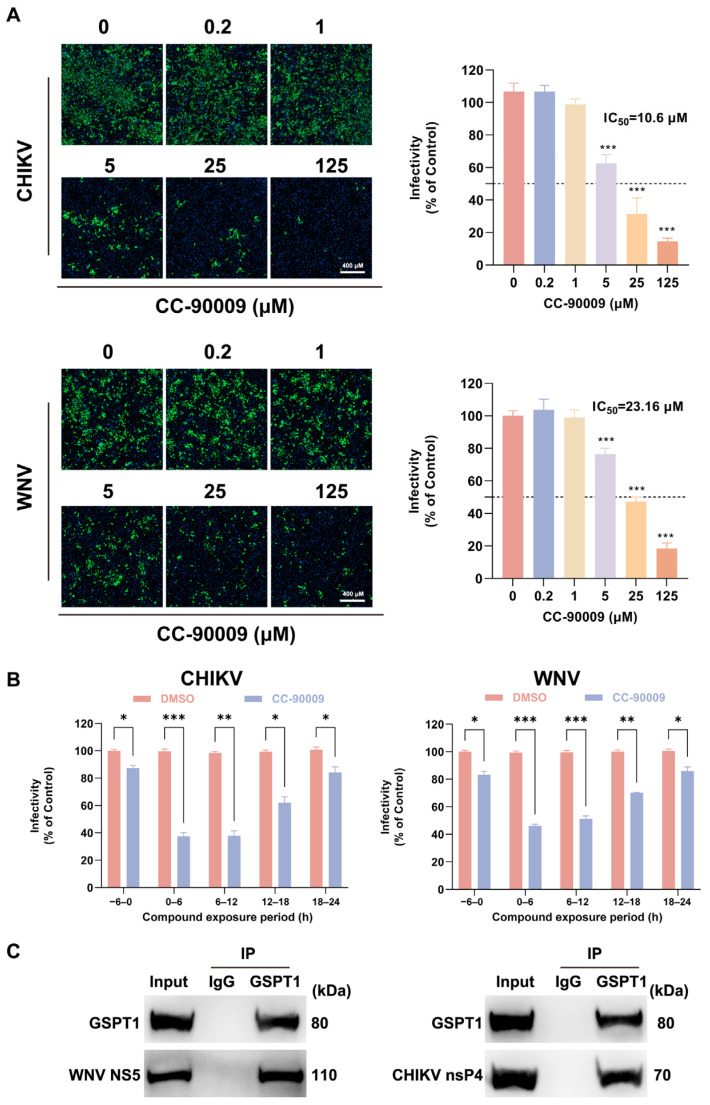
Broad-spectrum antiviral activity of CC-90009. (**A**) Dose–response analysis of CC-90009 in SH-SY5Y cells challenged with Chikungunya virus (CHIKV) or West Nile virus (WNV) (MOI = 1). Viral infectivity was quantified by IF assay. The dashed line denotes the 50% infection threshold; (**B**) CC-90009 was administered during the following time windows relative to infection: −6 to 0, 0–6, 6–12, 12–18, and 18–24 h. Viral infectivity was quantified by IF; (**C**) Co-IP of GSPT1 with WNV NS5 or CHIKV nsP4 in HEK-293T cells. Lysates were immunoprecipitated using an anti-GSPT1 antibody or non-specific IgG control, followed by Western blot analysis. Scale bar, 400 µM. * *p* < 0.05, ** *p* < 0.01 and *** *p* < 0.001, compared with control.

We next extended our analysis to determine whether the antiviral activity of CC-90009 extends to other mosquito-borne viruses. As shown in Figure 6A, CC-90009 treatment potently suppressed infections with both CHIKV and WNV in SH-SY5Y cells in a dose-dependent manner. Based on the IC_50_ values, CC-90009 exhibited the strongest antiviral activity against CHIKV (IC_50_ = 10.6 μM), followed by WNV (IC_50_ = 23.16 μM). Furthermore, time-window assays revealed that CC-90009 predominantly targets post-entry stages of both WNV and CHIKV infection (Figure 6B). Co-IP assays confirmed that GSPT1 physically interacts with NS5 of WNV and nsP4 of CHIKV (Figure 6C).

### 3.7. CC-90009 Confers Protection Against Lethal JEV Infection in Mice

**Figure 7:**
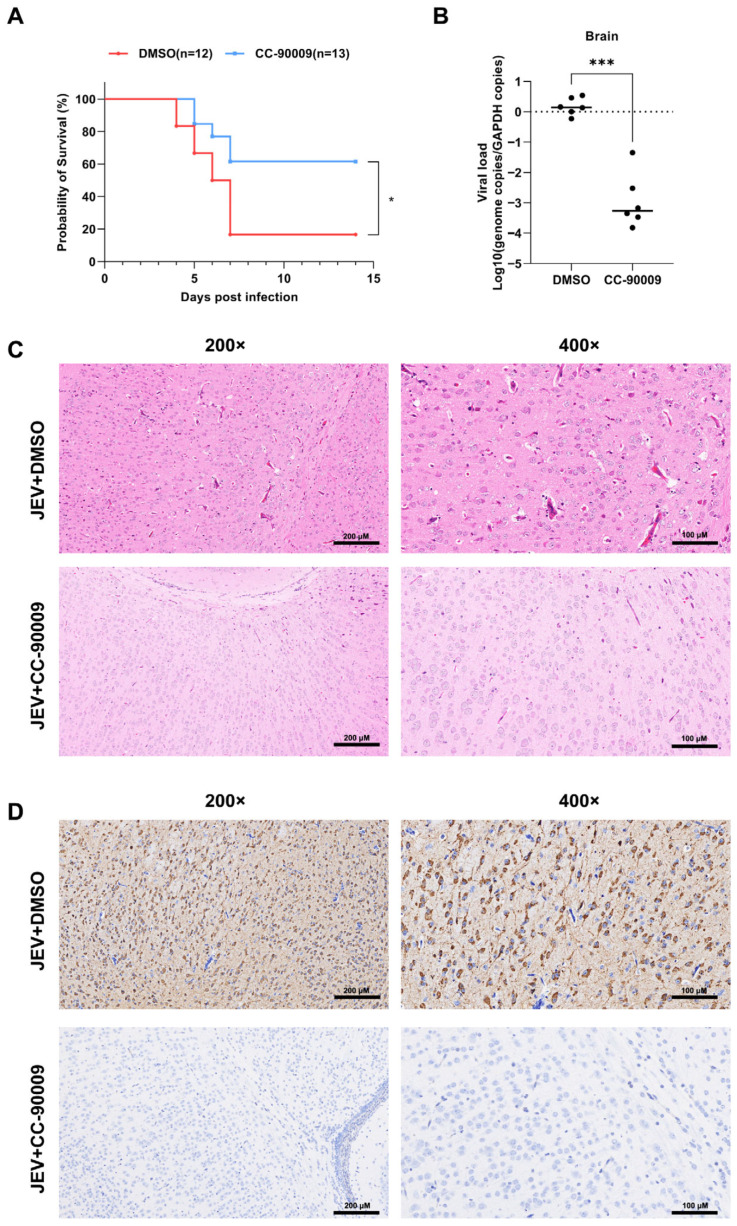
CC-90009 confers protection against lethal JEV infection in mice. (**A**) Kaplan–Meier survival curves of mice in the control and CC-90009-treated groups over a 14-day period post-infection. JEV + DMSO, n = 12, JEV + CC-90009, n = 13; (**B**) At 5 days post-infection, JEV RNA levels in mouse brain tissue were determined by RT-qPCR. Results are expressed as relative RNA genome copy number compared with controls. JEV + DMSO, n = 6, JEV + CC-90009, n = 6; (**C**) Mouse brain sections were detected using hematoxylin and eosin (H&E) staining; (**D**) Mouse brain sections were analysed by immunohistochemistry using an anti-JEV antibody. Results are representative of independent experiments. * *p* < 0.05 and *** *p* < 0.001, compared with control.

To evaluate the in vivo antiviral efficacy of CC-90009, we examined its protective effect against JEV infection in a murine model. Suckling Institute of Cancer Research mice were inoculated subcutaneously with a lethal dose of JEV and subsequently administered either DMSO or CC-90009. Survival was monitored daily. Mice in the JEV-infected, DMSO-treated group began exhibiting clinical signs—such as generalised tremors and unilateral limb paralysis—at three days post-infection. Mortality reached 83.3% (10/12) between four and seven days post-infection (Figure 7A). In contrast, CC-90009 treatment significantly alleviated symptoms and reduced the mortality rate to 38.5% (5/13).

Mice subjected to repeated CC-90009 treatment exhibited a markedly lower viral load in brain tissue than those in the DMSO-treated control group. (Figure 7B). Further analysis using hematoxylin and eosin (H&E) staining revealed that the CC-90009-treated group exhibited less severe histopathological damage in brain tissues compared with JEV-infected controls (Figure 7C). Additionally, immunohistochemical examination of mouse brain tissue showed high abundance of viral particles in the control group, whereas CC-90009 administration notably reduced the number of viral particles (Figure 7D).

## 4. Discussion

In this study, we found that CC-90009 acts as a broad-spectrum antiviral agent, inhibiting infections by JEV, WNV, and CHIKV in SH-SY5Y cells. Mechanistically, CC-90009 suppresses JEV translation processes and impairs viral genome replication by interfering with the protein levels of JEV NS5. In vivo experiments demonstrated that CC-90009 significantly protects suckling mice from JEV infection-induced mortality.

Here, we report for the first time that CC-90009 treatment induces the degradation of both GSPT1 and JEV NS5. The NS5 protein is the largest and most conserved enzyme encoded by flaviviruses. It is characterized by two functional domains: an N-terminal methyltransferase and a C-terminal RdRp [25]. The RdRps of flaviviruses are structurally similar: JEV and WNV share 70% identity with Zika virus, while Dengue virus (DENV)-2 and DENV-3 share 76% and 81% identity with Zika virus, respectively [26,27]. Based on the conservation of NS5 and its similarity between JEV and WNV, CC-90009 effectively inhibits both flaviviruses, most likely by targeting the NS5 protein of each virus. In the case of CHIKV, a member of the *Alphavirus* genus, its nsP4 protein also exhibits RdRp activity [28]; however, the precise mechanism of action of CC-90009, including its potential targeting of nsP4, remains to be elucidated. Collectively, these findings suggest the potential for developing CC-90009-based therapeutics against JE by targeting the JEV RdRp for degradation.

Our results demonstrated that CC-90009 treatment inhibited JEV infection in neuronal cells, with a significant reduction in NS5 protein levels. When proteasome function was inhibited by MG132, NS5 degradation was abrogated. Furthermore, CC-90009 failed to degrade NS5 upon CRBN inhibition. These findings collectively indicate that CC-90009 exerts its function by targeting NS5 for degradation via the proteasome pathway. However, we did not detect ubiquitination of NS5, suggesting that NS5 degradation may not occur through direct ubiquitination; instead, it might be indirectly targeted for proteasomal degradation together with GSPT1. By reprogramming the substrate specificity of the CRL4–CRBN E3 ligase, CC-90009 may convert JEV NS5 into a neosubstrate, thereby targeting it for degradation. Previous studies have also indicated that GSPT1 directly interacts with the viral polymerases of EBOV [20] and LASV [19]. Therefore, decreased NS5 abundance is expected to cause a genome-wide decline in JEV RNA synthesis.

Viral replication is highly dependent on the host translational apparatus [29]; consequently, multiple factors involved in translation initiation, elongation, and termination have emerged as promising targets for antiviral therapeutic development [30]. Human GSPT1 was initially identified as a key regulator of the G1-to-S phase transition [31]. Subsequent studies confirmed that it encodes a core component of the eukaryotic translation termination machinery, specifically functioning as the peptide chain release factor 3a [32]. The antiviral activity of CC-90009 against JEV appears to be both dependent on and specific to the downregulation of GSPT1. Similarly, compound 103, a targeted GSPT1 degrader, has been demonstrated to possess broad-spectrum antiviral activity [33]. Additionally, CC-90009 has entered clinical trials for the treatment of patients with acute myeloid leukaemia [14,18], which lays the groundwork for the application of GSPT1-deletion-based strategies in treating viral infections. Further exploration and research are required to elucidate the pharmacological properties, safety profile, and efficacy across different viral models to advance the therapeutic potential of GSPT1 degraders as pan-antiviral agents.

## Data Availability

The raw data supporting the conclusions of this article will be made available by the authors on request.

## Supplementary Materials

The following supporting information can be downloaded at: https://www.mdpi.com/article/10.3390/pharmaceutics17121524/s1, Figure S1: Chemical structure of CC-90009.

## Abbreviations

The following abbreviations are used in this manuscript:

JEV	Japanese encephalitis virus
NS5	Non-structural proteins
RdRp	RNA-dependent RNA polymerase
TPD	Targeted protein degradation
CRBN	Cereblon
CELMoD	Cereblon (CRBN) E3 ligase modulators
CRL4	Cullin4-RING E3 ligase complex
PROTAC	Proteolysis-targeting chimera
GSPT1	G1 to S phase transition 1
SH-SY5Y	Human neuroblastoma cell line SH-SY5Y
BHK-21	Baby hamster kidney cell line BHK-21
HEK-293T	Human embryonic kidney cell line HEK-293T
CHIKV	Chikungunya virus
WNV	West Nile virus
ATCC	American Type Culture Collection
IgG	Immunoglobulin
qRT-PCR	Quantitative real-time polymerase chain reaction
GAPDH	Glyceraldehyde 3-phosphate dehydrogenase
Co-IP	Co-immunoprecipitation
siRNA	Small interfering RNA
IF	Immunofluorescence
MOI	Multiplicity of infection
DMSO	Dimethyl sulfoxide

## References

[1] Zhu Y., Chen S., Lurong Q., Qi Z. (2023). Recent Advances in Antivirals for Japanese Encephalitis Virus. Viruses.

[2] Guo J., Mi Y., Guo Y., Bai Y., Wang M., Wang W., Wang Y. (2024). Current Advances in Japanese Encephalitis Virus Drug Development. Viruses.

[3] Edache S., Dixon A.L., Oliveira A.R.S., Cohnstaedt L.W., Mitzel D., Mire C.E., Cernicchiaro N. (2025). Mosquito vector competence for Japanese encephalitis virus: A systematic review and meta-analysis update. Parasites Vectors.

[4] Srivastava K.S., Jeswani V., Pal N., Bohra B., Vishwakarma V., Bapat A.A., Patnaik Y.P., Khanna N., Shukla R. (2023). Japanese Encephalitis Virus: An Update on the Potential Antivirals and Vaccines. Vaccines.

[5] Zhu Y., He Z., Qi Z. (2023). Virus-host Interactions in Early Japanese Encephalitis Virus Infection. Virus Res..

[6] Chugh P., Soni S., Ghanghas N., Kumar S., Mohan H. (2025). Comprehensive insights into Japanese encephalitis virus: From molecular characterization to advanced detection and vaccine strategies. Antivir. Res..

[7] Xu Q., Cao M., Song H., Chen S., Qian X., Zhao P., Ren H., Tang H., Wang Y., Wei Y. (2016). Caveolin-1-mediated Japanese encephalitis virus entry requires a two-step regulation of actin reorganization. Future Microbiol..

[8] Sarkar R., Chhabra S., Tanwar M., Agarwal N., Kalia M. (2024). Japanese encephalitis virus hijacks ER-associated degradation regulators for its replication. J. Gen. Virol..

[9] Kumar S., Verma A., Yadav P., Dubey S.K., Azhar E.I., Maitra S.S., Dwivedi V.D. (2022). Molecular pathogenesis of Japanese encephalitis and possible therapeutic strategies. Arch. Virol..

[10] Lu G., Gong P. (2013). Crystal Structure of the full-length Japanese encephalitis virus NS5 reveals a conserved methyltransferase-polymerase interface. PLoS Pathog..

[11] Hinterndorfer M., Spiteri V.A., Ciulli A., Winter G.E. (2025). Targeted protein degradation for cancer therapy. Nat. Rev. Cancer.

[12] Hansen J.D., Correa M., Nagy M.A., Alexander M., Plantevin V., Grant V., Whitefield B., Huang D., Kercher T., Harris R. (2020). Discovery of CRBN E3 Ligase Modulator CC-92480 for the Treatment of Relapsed and Refractory Multiple Myeloma. J. Med. Chem..

[13] Bonazzi S., d’Hennezel E., Beckwith R.E.J., Xu L., Fazal A., Magracheva A., Ramesh R., Cernijenko A., Antonakos B., Bhang H.C. (2023). Discovery and characterization of a selective IKZF2 glue degrader for cancer immunotherapy. Cell Chem. Biol..

[14] Surka C., Jin L., Mbong N., Lu C.C., Jang I.S., Rychak E., Mendy D., Clayton T., Tindall E., Hsu C. (2021). CC-90009, a novel cereblon E3 ligase modulator, targets acute myeloid leukemia blasts and leukemia stem cells. Blood.

[15] Snyder L.B., Neklesa T.K., Willard R.R., Gordon D.A., Pizzano J., Vitale N., Robling K., Dorso M.A., Moghrabi W., Landrette S. (2025). Preclinical Evaluation of Bavdegalutamide (ARV-110), a Novel PROteolysis TArgeting Chimera Androgen Receptor Degrader. Mol. Cancer Ther..

[16] Gough S.M., Flanagan J.J., Teh J., Andreoli M., Rousseau E., Pannone M., Bookbinder M., Willard R., Davenport K., Bortolon E. (2024). Oral Estrogen Receptor PROTAC Vepdegestrant (ARV-471) Is Highly Efficacious as Monotherapy and in Combination with CDK4/6 or PI3K/mTOR Pathway Inhibitors in Preclinical ER+ Breast Cancer Models. Clin. Cancer Res..

[17] Zhao L., Zhao J., Zhong K., Tong A., Jia D. (2022). Targeted protein degradation: Mechanisms, strategies and application. Signal Transduct. Target. Ther..

[18] Hansen J.D., Correa M., Alexander M., Nagy M., Huang D., Sapienza J., Lu G., LeBrun L.A., Cathers B.E., Zhang W. (2021). CC-90009: A Cereblon E3 Ligase Modulating Drug That Promotes Selective Degradation of GSPT1 for the Treatment of Acute Myeloid Leukemia. J. Med. Chem..

[19] Fang J., Pietzsch C., Witwit H., Tsaprailis G., Crynen G., Cho K.F., Ting A.Y., Bukreyev A., Saphire E.O., de la Torre J.C. (2022). Proximity interactome analysis of Lassa polymerase reveals eRF3a/GSPT1 as a druggable target for host-directed antivirals. Proc. Natl. Acad. Sci. USA.

[20] Fang J., Pietzsch C., Tsaprailis G., Crynen G., Cho K.F., Ting A.Y., Bukreyev A., de la Torre J.C., Saphire E.O. (2022). Functional interactomes of the Ebola virus polymerase identified by proximity proteomics in the context of viral replication. Cell Rep..

[21] He Z., Xia B., Zhao T., Zhao P., Ren H., Qi Z., Zhu Y. (2025). Clathrin-Independent Carriers/Glycosylphosphatidylinositol-Anchored-Protein-Enriched Endosomal Compartment Endocytic Pathway Is Critical for Enterovirus A71 Entry Into Human Oral Epidermoid Carcinoma KB Cells. J. Med. Virol..

[22] Zhu Y., Wang X., He Z., Zhao P., Ren H., Qi Z. (2023). Enterovirus 71 enters human brain microvascular endothelial cells through an ARF6-mediated endocytic pathway. J. Med. Virol..

[23] Liu Y.G., Chen Y., Wang X., Zhao P., Zhu Y., Qi Z. (2020). Ezrin is essential for the entry of Japanese encephalitis virus into the human brain microvascular endothelial cells. Emerg. Microbes Infect..

[24] Hellen C.U.T. (2018). Translation Termination and Ribosome Recycling in Eukaryotes. Cold Spring Harb. Perspect. Biol..

[25] Goh J.Z.H., De Hayr L., Khromykh A.A., Slonchak A. (2024). The Flavivirus Non-Structural Protein 5 (NS5): Structure, Functions, and Targeting for Development of Vaccines and Therapeutics. Vaccines.

[26] Murali A., Kumar S., Akshaya S., Singh S.K. (2023). Drug repurposing toward the inhibition of RNA-dependent RNA polymerase of various flaviviruses through computational study. J. Cell. Biochem..

[27] Malet H., Massé N., Selisko B., Romette J.L., Alvarez K., Guillemot J.C., Tolou H., Yap T.L., Vasudevan S., Lescar J. (2008). The flavivirus polymerase as a target for drug discovery. Antivir. Res..

[28] Tan Y.B., Lello L.S., Liu X., Law Y.S., Kang C., Lescar J., Zheng J., Merits A., Luo D. (2022). Crystal structures of alphavirus nonstructural protein 4 (nsP4) reveal an intrinsically dynamic RNA-dependent RNA polymerase fold. Nucleic Acids Res..

[29] den Boon J.A., Nishikiori M., Zhan H., Ahlquist P. (2024). Positive-strand RNA virus genome replication organelles: Structure, assembly, control. Trends Genet..

[30] Walsh D., Mohr I. (2011). Viral subversion of the host protein synthesis machinery. Nat. Rev. Microbiol..

[31] Liu C., Peng H., Chen P., Li Y., Deng Z., Li S., Yang T., Liu K., Wang Z., Liu L. (2025). GSPT1 degraders: Research progress, development strategies and challenges. Bioorg. Med. Chem..

[32] Cheng B., Wang Y., Hong Y., Zhou Y., Chen J., Zeng C. (2025). Research Progress in Targeting GSPT1: Molecular Glues, Bifunctional Degraders, and Antibody-Enabled Molecular Glues for Cancer Therapy. J. Med. Chem..

[33] Zhao N., Ho J.S.Y., Meng F., Zheng S., Kurland A.P., Tian L., Rea-Moreno M., Song X., Seo J.S., Kaniskan H. (2023). Generation of host-directed and virus-specific antivirals using targeted protein degradation promoted by small molecules and viral RNA mimics. Cell Host Microbe.

