# Supplementary material for: CC-90009, a Cereblon E3 Ligase Modulator, Exhibits Antiviral Efficacy Against JEV In Vitro and In Vivo via Targeted Degradation of GSPT1 and Viral NS5 Protein
Source: Pharmaceutics. 2025 Nov 27;17(12):1524. doi: 10.3390/pharmaceutics17121524 (PMC12736548; doi:10.3390/pharmaceutics17121524)
Supplement: Supplementary file 1 [file pharmaceutics-17-01524-s001.zip › pharmaceutics-3964640-supplementary.pdf]

Supplemental information

CC-90009, a Cereblon E3 Ligase Modulator, Exhibits Antiviral Efficacy Against JEV In Vitro and In Vivo via Targeted Degradation of GSPT1 and Viral NS5 Protein

Zhiwei He [1,†], Yibo Chen [1,†], Binghui Xia [1], Zimeng Cheng [1], Ping Zhao [1,2,3], Zhongtian Qi [1,2,3,*] and Yongzhe Zhu [1,2,3,*]

1 Department of Microbiology, Faculty of Naval Medicine, Naval Medical University, Shanghai 200433, China; hezw@smmu.edu.cn (Z.H.); miraclechen03@163.com (Y.C.); xbhnjucpu@smmu.edu.cn (B.X.); 13582818881@163.com (Z.C.); pnzhao@163.com (P.Z.)
2 Shanghai Key Laboratory of Medical Bioprotection, Second Military Medical University, Shanghai 200433, China
3 Key Laboratory of Biological Defense, Ministry of Education, Second Military Medical University, Shanghai 200433, China
* Correspondence: qizt@smmu.edu.cn (Z.Q.); zhuyz@smmu.edu.cn (Y.Z.)
† These authors contributed equally to this work.

Materials and methods

Chemicals and antibodies

CC-90009 2-(4-chlorophenyl)-N-((2-(2,6-dioxopiperidin-3-yl)-1-oxoisoindolin-5-yl) methyl)-2,2-difluoroacetamide, is a cereblon E3 ligase modulating drug (CELMoD) with antiproliferative and proapoptotic activity against acute myeloid leukemia (AML) (Figure below). CC-90009 derives its anti-AML efficacy through degradation of GSPT1 (G1 to S phase transition 1) and is currently in phase 1 clinical trials for relapsed/refractory acute myeloid leukemia. In our study, CC-90009 demonstrated potent antiviral activity against JEV (IC_{50} = 3.847 μM), CHIKV (IC_{50} = 10.6 μM), and WNV (IC_{50} = 23.16 μM), coupled with low cytotoxicity (CC_{50} = 333.9 μM), indicating a high therapeutic index. Detailed pharmacological data from other disease contexts, particularly in acute myeloid leukemia, are well-documented in the references [14] and [18].

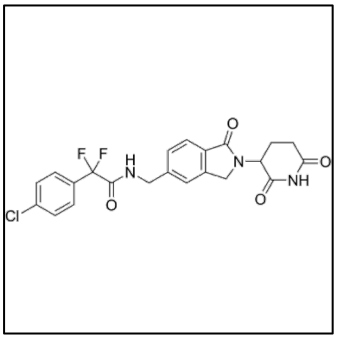

Figure S1. Chemical structure of CC-90009.
